# Biosynthesis of novel non-proteinogenic amino acids β-hydroxyenduracididine and β-methylphenylalanine in *Escherichia coli*

**DOI:** 10.3389/fbioe.2024.1468974

**Published:** 2024-10-09

**Authors:** Rosemary Gillane, Dara Daygon, Zeinab G. Khalil, Esteban Marcellin

**Affiliations:** ^1^ Australian Institute for Bioengineering and Nanotechnology, University of Queensland, Brisbane, QLD, Australia; ^2^ ARC Centre of Excellence in Synthetic Biology, University of Queensland, Brisbane, QLD, Australia; ^3^ Queensland Metabolomics and Proteomics Facility, University of Queensland, Brisbane, QLD, Australia; ^4^ Institute of Molecular Biosciences, University of Queensland, Brisbane, QLD, Australia

**Keywords:** non-proteinogenic amino acids, *E. coli*, β-methylphenylalanine, enduracididine, β-hydroxyenduracididine, *Streptomyces*, heterologous expression

## Abstract

Non-proteinogenic amino acids (npAAs) are valuable building blocks for the development of advanced pharmaceuticals and agrochemicals. The surge in interest in their synthesis is primarily due to the potential to enhance and diversify existing bioactive molecules. This can be achieved by altering these bioactive molecules to improve their effectiveness, reducing resistance compared to their natural counterparts or generating molecules with novel functions. Traditional production of npAAs in native hosts requires specialized conditions and complex cultivation media. Furthermore, these compounds are often found in organisms that challenge genetic manipulation. Thus, the recombinant production of these npAAs in a model organism like *Escherichia coli* paves the way for groundbreaking advancements in synthetic biology. Two synthetic operons, comprising of five heterologous proteins were genomically integrated into *E. coli* for the synthesis of npAAs β-methylphenylalanine (BmePhe), β-hydroxyenduracididine (BhEnd), and enduracididine (End). Proteomic and metabolomic analysis confirmed production of these compounds in E. coli for the first time. Interestingly, we discovered that the exogenous addition of pathway precursors to the *E. coli* system enhanced the yield of BmePhe by 2.5 times, whereas it concurrently attenuated the production of BhEnd and End, signifying a selective precursor-dependent yield enhancement. The synthetic biology landscape is broadened in this study by expanding the repertoire of amino acids beyond the conventional set of 22 standard proteinogenic amino acids. The biosynthesized npAAs, End, BhEnd, and BmePhe hold promise for engineering proteins with modified functions, integrating into novel metabolites and/or enhancing biological stability and activity. Additionally, these amino acids’ biological production and subsequent purification present an alternative to traditional chemical synthesis methods, paving a direct pathway for pharmacological evaluation.

## 1 Introduction

Proteins, the architects of life’s complexity, are traditionally composed of 22 standard amino acids. Yet nature extends beyond this limited set, encompassing a diverse array of non-proteinogenic amino acids (npAAs) that are not customarily encoded by the genetic code. These non-standard amino acids are pivotal in numerous biological functions and hold immense potential in pharmaceuticals, agriculture, and bioengineering. They play critical roles in a variety of biological processes and possess promising applications. Furthermore, npAAs represent a frontier for innovative chemical exploration, poised to enhance the versatility of biological systems.

NpAAs include molecules that are precursors in biosynthesis, post-translationally modified, specially synthesized for non-ribosomal peptide synthesis (NRPS) or hybrid polyketide synthesis (PKS), and chemically synthesized. NpAAs can be found in bacteria, fungi, and plants and can act as secondary metabolites (Fichtner et al., 2017) or be incorporated into large secondary metabolic scaffolds typically via NRPS (Felnagle et al., 2008; Xu et al., 2020). However, technical challenges in producing them in large quantities have limited our capacity to harness their full potential.

The vast repertoire of amino acid structures in nature is astounding, with hundreds of unique variants across many organisms (Yamane et al., 2010; Ding et al., 2020). NpAAs have found multiple applications in biopharmaceutical innovation. D-amino acids are commonly utilized in the formulation of biologics due to their enhanced resistance to degradation (Wang L. et al., 2022). This property significantly contributes to prolonging the stability and efficacy of drugs. Beta-modified amino acids are also used in peptide-based drugs to change how they work in the body and to make them more effective (Ren et al., 2019).

However, there are numerous challenges when it comes to synthetic biology and the aim of harnessing these unique compounds. Many organisms that naturally produce these npAAs are not suitable for laboratory manipulation for increased production due to the absence of refined genetic engineering techniques. Additionally, the complexity of isolating and purifying these novel metabolites, coupled with the obstacles in maintaining such organisms under standard lab conditions, further complicates their use.

*Streptomyces* are a group of bacteria known for their prolific production of bioactive secondary metabolites and are substantial contributors to novel npAAs. However, many *Streptomyces* species are hard to culture and difficult to engineer. As a result, creating these npAAs from recombinant proteins in model organisms has huge potential for downstream applications, including purification for activity testing and medicinal use. Commonly, commercially produced amino acids are cultivated by large-scale fermentation with sugar substrates (Industrial Production of Amino Acids by Microorganism and Fermentation, 2023; D’Este et al., 2018). The extensive understanding of *E. coli*, coupled with its ease of fermentation and the ability to engineer it for enhanced production, renders this organism a popular choice for industrial cultivation.

Here, we engineered *E. coli* to produce two important npAAs: β-methylphenylalanine (BmePhe) and β-hydroxyenduracididine (BhEnd). BmePhe is synthesized from phenylpyruvate as a starting product and methylated by MppJ at the β-carbon position. This reaction is followed by a transaminase reaction by the promiscuous TyrB to yield BmePhe and convert L-glutamate to 2-oxoglutarate (Huang et al., 2009; Zou et al., 2014). The BhEnd pathway utilizes L-arginine and contains four enzymatic steps to yield the final product. MppP oxygenates arginine by replacing the amino group with a carboxyl group (Han et al., 2015), and MppR (Burroughs et al., 2013) cyclizes the compound to create the signature side chain. MppQ transaminases the compound to construct L-enduracididine while converting L-alanine to pyruvate to replenish the amine group. Although L-enduracididine is an npAA in its own right, MppO oxygenates the L-enduracididine at the β-carbon position for BhEnd production (Haltli et al., 2005).

These npAAs were selected as BmePhe displays an array of medicinal properties, which include being antiarthritic (Ren et al., 2019), antinociceptive (Kovács et al., 2012), and neuroprotective against Parkinson’s disease (Feng et al., 2020). It is also found in larger secondary metabolites including the antibiotic bottromycin (Franz et al., 2021), the antibiotic mannopeptimycin (Singh et al., 2003), and the isoleucyl-tRNA synthetase inhibitor SB-203208 (Houge-Fkydrych et al., 2000).

The enduracididine family of molecules is known for its integration into large bioactive molecules, including antibiotics enduracidin (Yin and Zabriskie, 2006), minosaminomycin (Hamada et al., 1974), mannopeptimycin (Singh et al., 2003), and teixobactin (Ling et al., 2015) but has no reports on potential activity as a stand-alone compound. Although the biosynthetic process of BhEnd is clear, the chemical synthesis has posed a challenge and has been attempted with varying success by multiple groups (Schwörer and Oberthür, 2009; Olivier and Van Nieuwenhze, 2010; Lin et al., 2016a; 2016b). To investigate heterologous production of the npAAs, synthetic operons containing the npAA pathways were introduced into *E. coli*. The data presented here are pivotal for understanding the heterologous biosynthesis of npAA in recombinant organisms and serve to extend the chemical potential for synthetic biology applications.

## 2 Results

### 2.1 Incorporation of npAAs into biological systems results in a growth burden, reflecting the additional metabolic strain they impose

**FIGURE 1:**
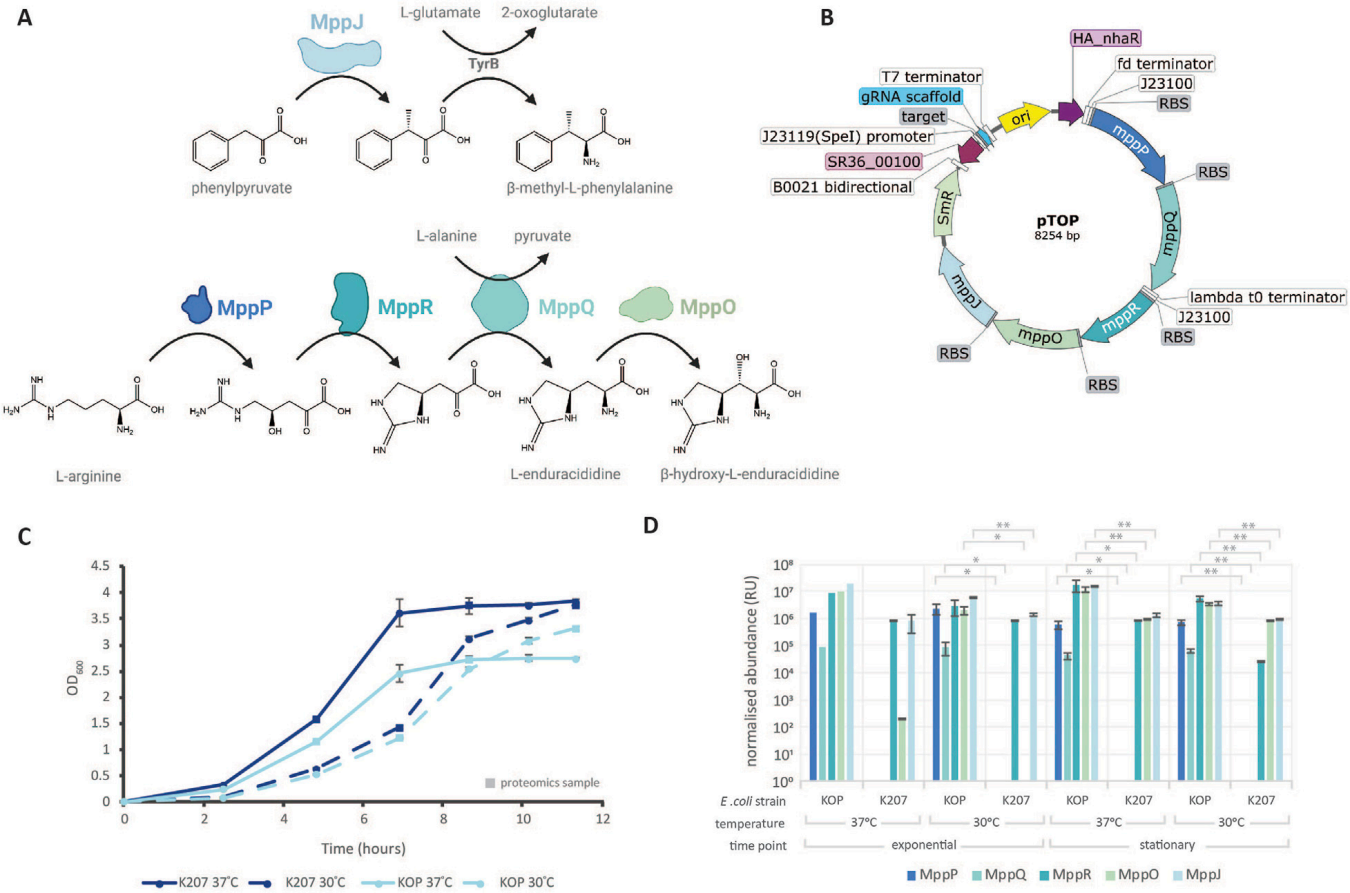
Synthetic operon expression in *E. coli* KOP. **(A)** Chemical pathway for the synthesis of β-methylphenylalanine and β-hydroxyenduracididine. Introduced *Streptomyces* proteins are depicted in color, and native *E. coli* enzymes are in gray, made with BioRender. **(B)** Plasmid map of pTOP for the CRISPR introduction of non-proteinogenic amino acids, made in SnapGene. **(C)** Growth curves of parental *E. coli* K207 and engineered *E. coli* KOP at 37°C and 30°C. OD_600_ measurements were taken from biological triplicates. Data points represent the average ± S.D. N = 3. Time points processed for proteomics are depicted as squares. **(D)** Normalized protein abundance of introduced proteins MppR, MppQ, MppR, MppO, and MppJ detected via LC–MS/MS for the parental *E. coli* K207 and engineered *E. coli* KOP. N = 3 (except N = 1 *Escherichia coli* KOP, 37°C, exponential). *p*-values * = < 0.05 and ** = < 0.01.

*Escherichia coli* KOP was developed for the constitutive production of MppP, MppQ, MppR, MppO, and MppJ from synthetic operons. These genes were integrated into the chromosome of *E. coli* K207-3 (Murli et al., 2003) (Supplementary 1), which was chosen as a starting strain due to the presence of an internal 4′-phosphopantetheinyl transferase sfp protein (PPTase) that could be used to support NRPS and PKS expression systems in the future. Our results demonstrate that the expression of the five proteins responsible for the synthesis of β-methylphenylalanine and β-hydroxyenduracididine places a significant burden on *E. coli* KOP. This is evident from the data presented in Figures 1A–C. The growth rates and doubling times (DT) for K207 and KOP at 37°C were 1.070 h^−1^ (DT 38.9 min) and 0.997 h^−1^ (DT 41.7 min), respectively. At 30°C, K207 and KOP showed growth rates and doubling time of 0.918 h^−1^ (DT 45.3 min) and 0.793 h^−1^ (DT 47.6 min), respectively. Significant differences in growth rates were observed when comparing KOP and K207 at 37°C (*p* < 0.005) and 30°C (p < 0.05). Each time point also showed a significant difference (p< 0.05) in OD_600_ at all time points greater than 0 h. Interestingly, at 37°C, *E. coli* KOP reached an optical density (OD) maximum ¾ of *E. coli* K207. Unsurprisingly, strains cultured at 30°C also showed a decrease in the growth rate but grew toward the same maximal OD_600_ as K207 at 37°C.

### 2.2 Proteomics confirms the production of engineered pathways

Five proteins, organized into two distinct pathways as illustrated in Figure 1A, were engineered into *E. coli* KOP. Compared to the *E. coli* K207 parental strain, *E. coli* KOP demonstrated a statistically significant increase in protein expression, as detailed in Figure 1B. There was no decrease in protein expression when strains were cultured at 37°C compared to 30°C, proving that *E. coli* growth conditions can maintain the folding of native *Streptomyces* proteins, usually cultured at 30°C. There was also no difference in protein concentration in the stationary or exponential phase. This suggests that MppJ, MppQ, MppR, MppP, and MppO may be constitutively expressed throughout the fermentation and independent of the growth phase, aligning with our synthetic constructs’ design.

**FIGURE 2:**
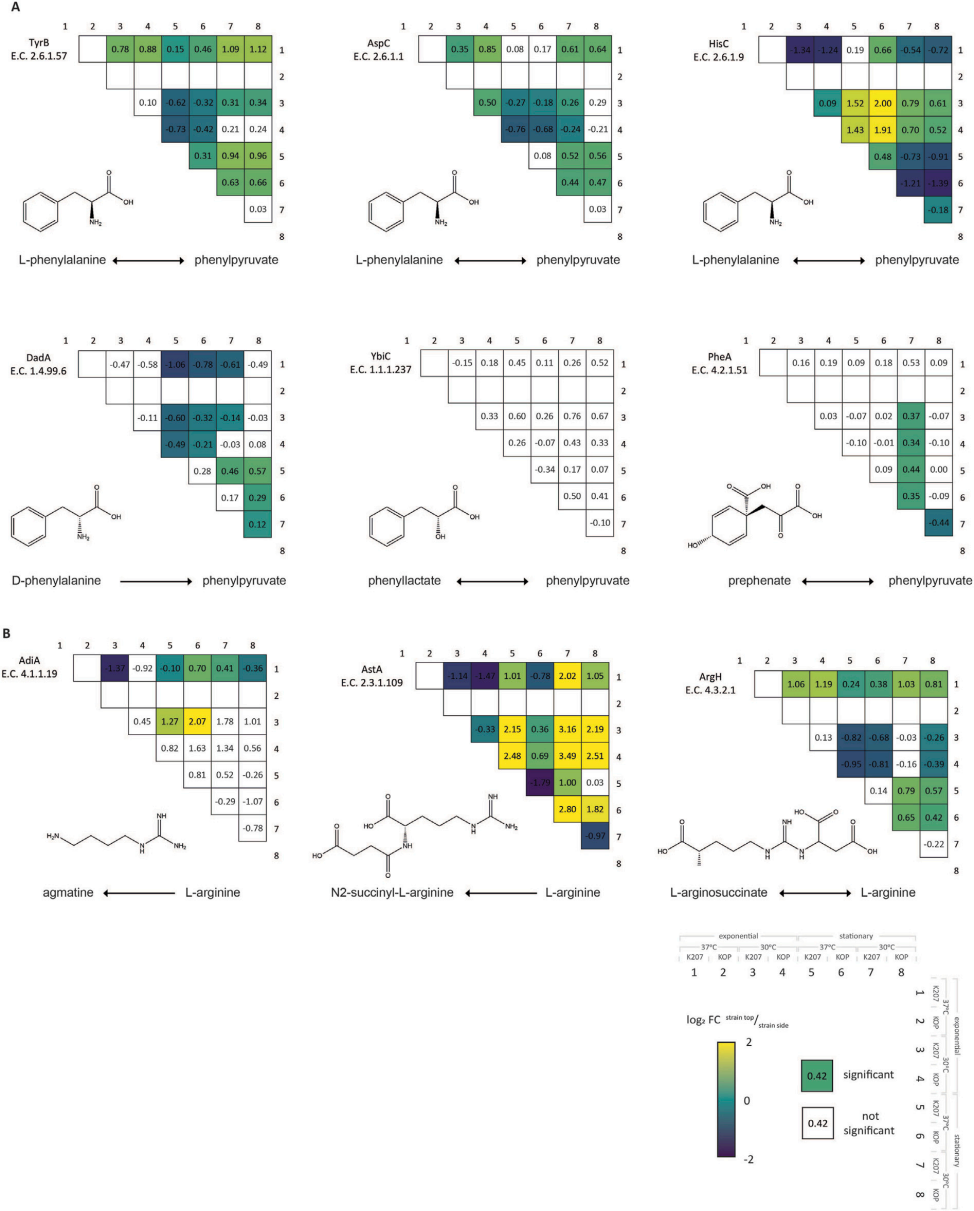
Differential expression of proteins that synthesize or deplete phenylpyruvate **(A)** and arginine **(B)** under varying conditions. Each value in the square represents the log_2_ FC of the top condition/side condition. Squares that are colored are significantly different. Condition 1: *E. coli* K207, 37°C, and exponential phase; condition 2: *E. coli* KOP, 37°C, and exponential phase; condition 3: *E. coli* K207, 30°C, and exponential phase; condition 4: *E. coli* KOP, 30°C, and exponential phase; condition 5: *E. coli* K207, 37°C, and stationary phase; condition 6: *E. coli* KOP, 37°C, and stationary phase; condition 7: *E. coli* K207, 30°C, and stationary phase; condition 8: *E. coli* KOP, 30°C, and stationary phase.

Among the proteins influencing the internal pools of arginine or phenylpyruvate, only a few selected proteins show significant differences in expression between the *E. coli* KOP and K207 strains under each tested condition, as detailed in Figure 2. There are three enzymes, namely tyrosine aminotransferase (TyrB), aspartate aminotransferase (AspC), and histidinol-phosphate aminotransferase (HisC), which are capable of converting L-phenylalanine to phenylpyruvate via an aminotransferase reaction. TyrB shows a significant difference at the stationary phase at 37°C, with cultures containing 20% more TyrB in KOP than K207. During the exponential phase at 30°C, AspC has a log_2_ FC of 0.50, and HisC shows variable changes between KOP and K207 at different conditions. D-amino acid dehydrogenase (DadA) facilitates the direct conversion of D-phenylalanine to phenylpyruvate and is significantly increased in KOP at 30°C in the stationary phase. The conversion with prephenate was also only significantly different at this condition with more bifunctional chorismate mutase/prephenate dehydratase (PheA) in *E. coli* K207. Furthermore, the reversible phenyllactate to phenylpyruvate reactions have no significant differences between any conditions or strains. For the most part, the two strains were relatively consistent around phenylpyruvate at different conditions tested, which further supports cultivation at the *E. coli* optimal temperature of 37°C.

### 2.3 β-Methylphenylalanine and β-hydroxyenduracididine can be produced in *Escherichia coli*

**FIGURE 3:**
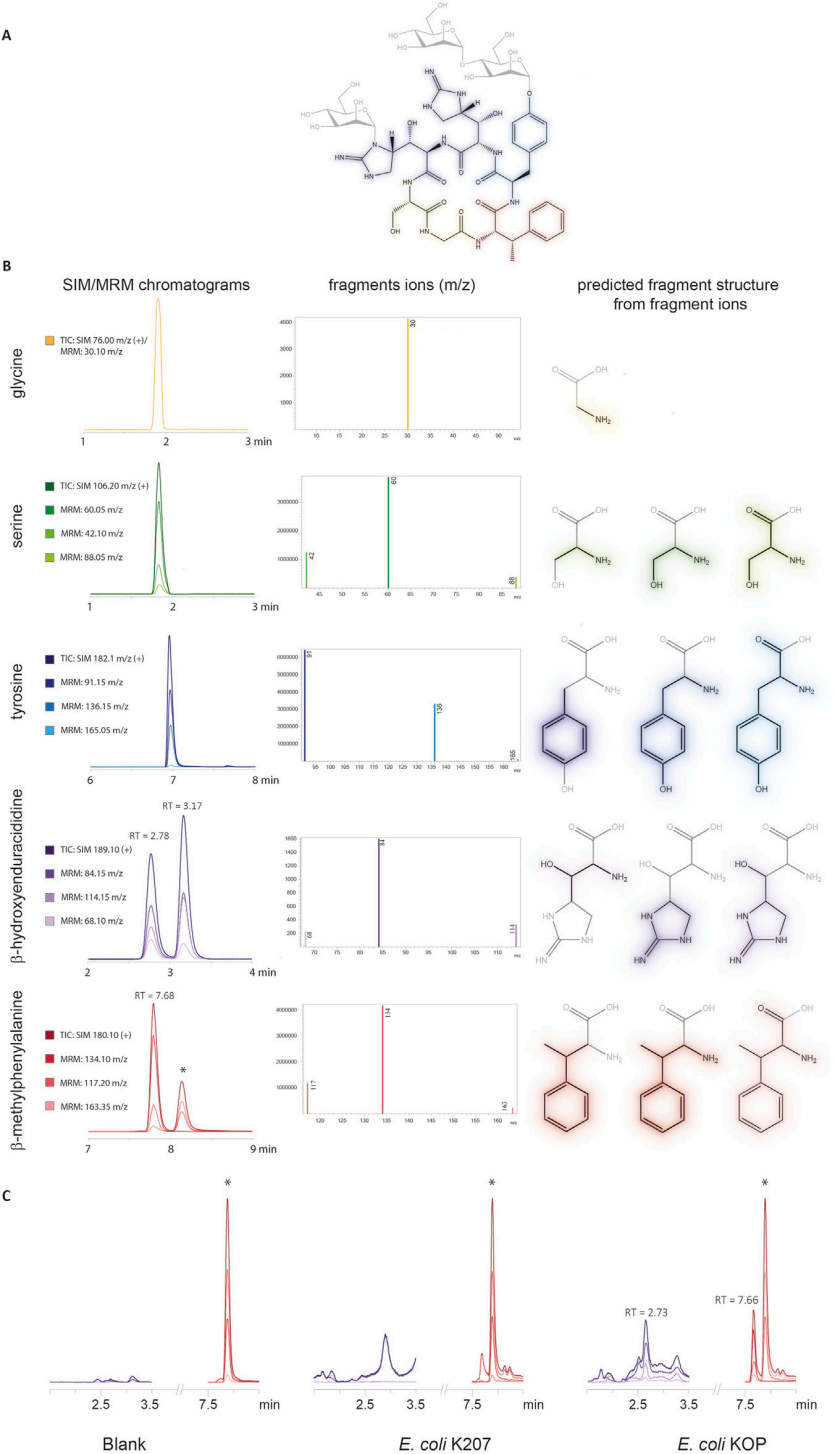
LC–MS/MS analysis of standards generated from mannopeptimycin acid hydrolysis. **(A)** Mannopeptimycin-α structure. **(B)** LC–MS/MS peaks for the SIM and MRM for each amino acid found in the mannopeptimycin structure. Each peak has the fragment ions identified and the corresponding fragment structure. **(C)** Chromatograms of the MRM methods for the detection of BhEnd and BmePhe in the parental *E. coli* K207 and engineered *E. coli* KOP strain. * denotes contaminating peak.

Acid hydrolysis of mannopeptimycin α standard, depicted in Figure 3A, resulted in the release of the amino acids from the cyclic hexapeptide which was analyzed via LC–MS/MS. The presence of tyrosine, serine, and glycine was confirmed against standards for each compound, and the multi-reaction monitoring (MRM) method was also confirmed against these products, as shown in Figure 3B. Single ion monitoring (SIM) for BhEnd (189.10 m/z) resulted in two peaks at 2.7 and 3.2 min and BmePhe (180.10 m/z) resulted in one peak at 7.7 min. The two peaks for BhEnd correspond to the release of D-β-hydroxyenduracididine and L-β-hydroxyenduracididine for the mannopeptimycin-α standard but have not been assigned to each of the peaks at 2.7 and 3.2 min, as shown in Figure 3B. The most common daughter fragments found for BhEnd were 84.15, 114.15, and 68.10 m/z, and the most common for BmePhe were 134.10, 117.20, and 163.35 m/z.

The MRM method detected the presence of BmePhe and BhEnd parent mass ions and fragment mass ions in *E. coli* KOP, as shown in Figure 3C. Although there are some peaks at a similar retention time in *E. coli* K207, the peaks lack the characteristic daughter ion fragments that are seen in the standards and are likely not attributable to the npAAs.

### 2.4 Increasing pathway precursors increases npAA production

Based on our relative comparison, the levels of BhEnd and BmePhe were quite low in *E. coli* KOP. Specific pathway precursors were incorporated into the culture media to stimulate the production through the engineered pathways. This included a mixture of 2 mM L-phenylalanine (serving as an alternative to phenylpyruvate) and 2 mM L-glutamic acid, which is intended to enhance the activity of methyltransferase MppJ and aminotransferase TyrB, respectively. Additionally, the BhEnd pathway was boosted by adding 2 mM of L-arginine and 2 mM of L-alanine. Furthermore, *E. coli* KOP cultures were also grown in media enriched with glucose to potentially augment overall carbon metabolism.

**FIGURE 4:**
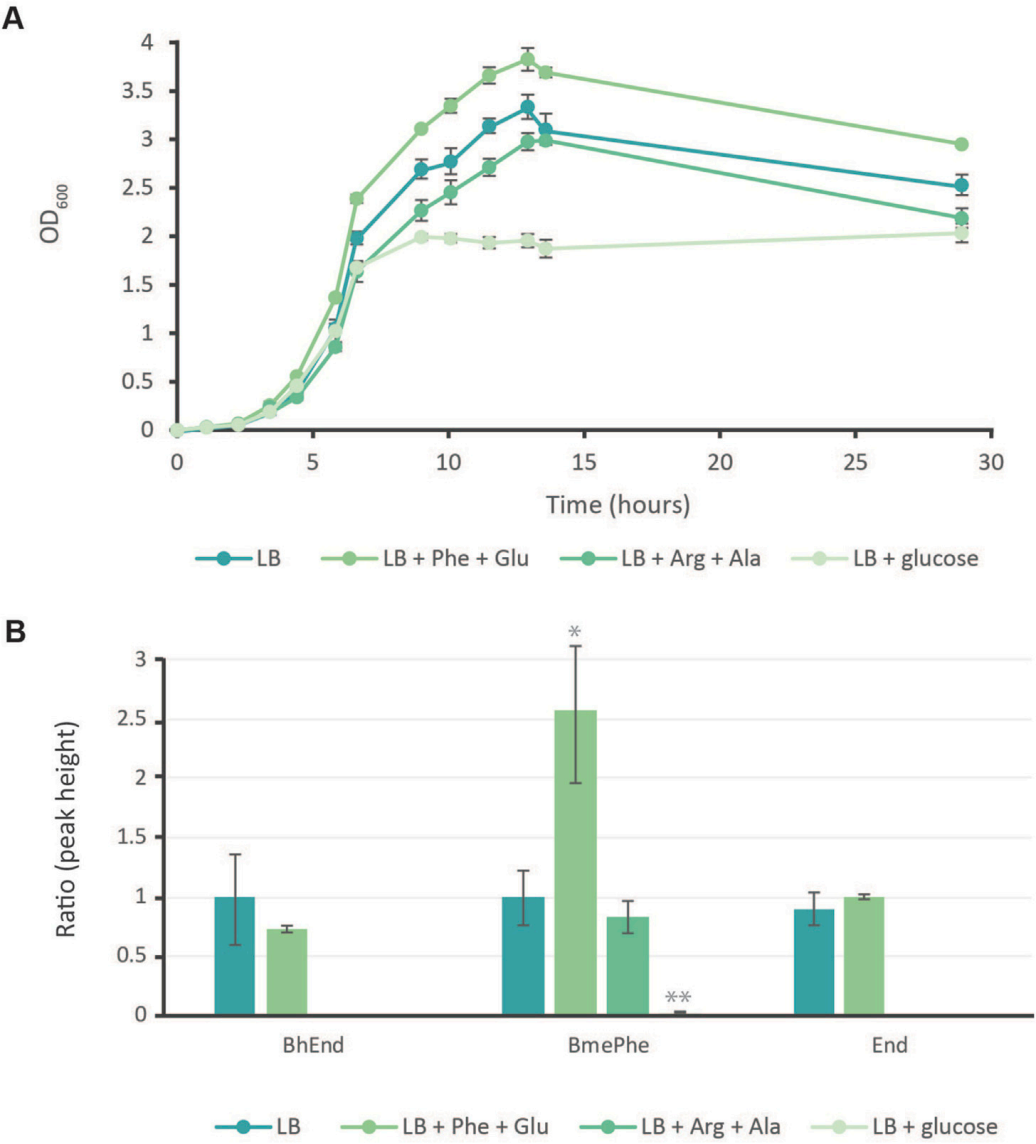
Growth of *E. coli* KOP with precursor additives to enhance the production of BmePhe and BhEnd. **(A)** Growth curves of *E. coli* KOP in LB with Phe: phenylalanine and Glu: glutamic acid, Arg: arginine and Ala: alanine, or with glucose. Each point represents the average of biological triplicates +/− the standard deviation. **(B)** The ratio of npAAs (BhEnd: β-hydroxyenduracididine; BmePhe: β-methylphenylalanine; and End: enduracididine) produced compared to strains grown in LB only. Each bar represents the average of triplicates +/− the standard deviation. *p*-values * = > 0.05, ** = > 0.01.

During the exponential growth phase, cultures grown in LB, LB supplemented with arginine and alanine, or LB supplemented with glucose exhibited similar growth rates, as shown in Figure 4A. However, cultures in LB enriched with phenylalanine and glutamic acid displayed a marginally faster growth rate. In the linear growth phase, the LB and LB cultures supplemented with amino acids maintained consistent growth rates. Specifically, phenylalanine and glutamic acid additions achieved an OD close to 4, LB alone reached an OD of 3.4, and the arginine and alanine mixture achieved an OD of 3. Notably, cultures with glucose supplementation showed a surprising pattern, barely progressing into linear growth and attaining a maximum OD of only 2. Although this significantly reduced OD, these glucose-supplemented cultures remained viable for up to 30 h into fermentation, whereas other cultures had ceased to survive.

BhEnd and BmePhe were detected again in standard LB culturing and in cultures with phenylalanine and glutamic acid, as shown in Figure 4B. BmePhe was also detected in cultures with arginine and alanine, but no BhEnd was confidently identified. When glucose levels were artificially elevated, the production of npAAs was effectively eliminated. Although a minimal quantity of BmePhe was detectable, its production was drastically reduced, being 50 times lower than that in the LB medium. Notably, BhEnd production reached its peak in cultures that were solely supplemented with LB.

In the presence of phenylalanine and glutamic acid, relative levels of BhEnd are 75% of the levels observed in LB-only fermentations. However, this difference in concentration was not statistically significant. The most drastic changes in production were seen for BmePhe; supplying the precursors for the pathway with as little as 2 mM of phenylalanine and glutamic acid increased production by 2.5 times, but the addition of arginine and alanine resulted in no difference in the amount of BmePhe produced. Enduracididine peaks were also found in LB cultures and cultures fed with phenylalanine and glutamic acid. Although not detected against an MRM method, the peaks that contain End were verified by the daughter fragment ion patterns of a 173 m/z SIM against those of the standard (Supplementary 5).

As we saw an unexpected decrease in production for strains that were grown in alanine and arginine, we decided to further interrogate the effect of these amino acids on the growth of our *E. coli* strains. *E. coli* K207 and *E. coli* KOP were grown in 20 mM of each amino acid (Supplementary 4). For both strains, maximal growth was increased by the presence of alanine. For cultures containing arginine, there is a clear increase in the lag phase and a decrease in maximal growth for both strains tested. For *E. coli* K207, the maximal growth was recovered slightly by the addition of alanine; however, this was not the case for *E. coli* KOP. The presence of both amino acids further worsened the overall growth of the bacteria.

## 3 Discussion

*Streptomycetes* are extraordinary producers of natural products, ranging in size from small npAAs to larger peptides in the 1,000s of Da. *Streptomyces hygroscopicus* produces npAAs, namely, β-hydroxyenduracididine and β-methylphenylalanine, that have not been researched in detail beyond the structural and functional assays of proteins in the biosynthetic pathway (Burroughs et al., 2013; Han et al., 2015; 2018; Vuksanovic, 2020). Although these compounds would enable a broader synthetic biology toolbox, manipulation and production of compounds in *Streptomyces* can be challenging. Metabolic engineering in *E. coli* is a viable alternative as these strains are reliable, easy to culture, scalable, and have streamlined purification processes.

*Escherichia coli* KOP has extraordinary potential for further downstream applications. To our knowledge, this is the first time that enduracididine, β-hydroxyenduracididine, or β-methylphenylalanine has been produced in any host strain outside of the naturally producing *Streptomyces* species. Although unquantified, we saw a progressive increase in the production of BmePhe throughout experimentation. Feeding cultures using just 2 mM of phenylalanine and glutamic acid resulted in a 2.5-fold increase in the compound. The production of BhEnd was seen sporadically in the conditions tested, and when detected, it was rarely seen above baseline concentrations, and more often, we saw the accumulation of End.

Based on the LCMS results throughout these studies, there appears to be less flux through the BhEnd pathway compared to the BmePhe pathway. This could be because the BmePhe pathway only has two proteins, one of which is heterologously expressed, compared to BhEnd’s four proteins. All of the proteins were codon optimized for expression in *E. coli*, and all proteins expressed in operons that were also optimized. From the pathway proteomics’ shown in Figure 1D, we also clearly see that each protein is expressed; however, these proteins are expressed in ratios which are inconducive for maximum metabolite production. There has been a magnitude of studies that have altered the ratios of enzymes to increase the product output and to reduce the accumulation of toxic intermediates (Domínguez et al., 1994; Jiang et al., 2020; Wightman et al., 2020). If high production of BhEnd is the desired outcome for future experimentation, it may be worthwhile to return to a standard *E. coli* strain and express each MppP, MppQ, MppR, and MppO under individual promoters with varying strengths and similar RBS transcription rates or put all four proteins under on high-expressing constitutive promoter with varying RBS rates. With plasmid libraries and high-throughput screening, finding a strain capable of elevated BhEnd production should be easy. It would be interesting to study the proportion of protein and/or mRNA of MppP, MppQ, MppR, and MppO in *S. hygroscopicus* (as theoretically, this strain has evolved for optimal BhEnd production) and then reverse engineer these back into *E. coli*. Of course, these processes could also be completed without MppO for End optimization.

Although the lack of BhEnd could be due to the lack of pathway optimization, BhEnd production could be toxic to *E. coli*. We saw no production with the inclusion of arginine and alanine, which are compounds that should theoretically increase the amount of BhEnd (Figure 4). Comparisons of *E. coli* KOP against K207 with arginine showed a decrease in both strains, whereas alanine alone increased the growth (Supplementary 4). The exact reason for the drastic reduction in the bacterial growth is unknown; engineered *E. coli* strains can produce upward of 10 g/L (Ginesy et al., 2015), so it is unlikely that the presence of arginine itself is toxic to *E. coli*. For *E. coli* K207, when cultured with both amino acids, the presence of alanine seemed to negate some of the adverse effects seen by arginine. This, however, was not seen for *E. coli* KOP, and the presence of alanine further decreased the maximal growth of the strain.

In a larger context, the npAAs produced from *E. coli* KOP could be used for many synthetic biological applications, including reengineering tRNAs to make novel proteins, making stable biologics, heterologous production of NRPSs, and novel natural product synthesis. *E. coli* KOP could be used as a starting point for further engineering secondary metabolites into chassis strains, such as bottromycin (Franz et al., 2021), SB-203207 (Houge-Fkydrych et al., 2000) (which contains BmePhe), enduracidin (Yin and Zabriskie, 2006), or minosaminomycin (Hamada et al., 1974) (which contain BhEnd/End). Mannopeptimycin incorporates BhEnd and BmePhe (Singh et al., 2003), whereas teixobactin presents a case in which analogs N-methylphenylalnine and L-*allo*-enduracididine are incorporated into the antibiotic (Shukla et al., 2022). Mannopeptimycin and teixobactin are antibiotics targeting Gram-positive bacteria by acting against the lipid II membrane. The more exciting possibilities lie in utilizing this *E. coli* strain to discover novel secondary metabolites. For instance, many *Streptomyces* strains have biosynthetic gene clusters that contain pathways to make either BmePhe or BhEnd but have unidentified small molecules.

Overall, the engineering of *E. coli* to produce β-hydroxyenduracididine, enduracididine, and β-methylphenylalanine has been successful. This is the first instance of these npAAs being produced recombinantly. BmePhe production could be used for large-scale fermentation and purification that could replace chemical synthesis; the production of BmePhe is important as it has a wide range of pharmacological uses. BhEnd and End have never been produced for potential purification and could have a wide range of benefits yet to be tested. Furthermore, this strain has a strong potential to be a chassis for producing or identifying secondary metabolites with industrial relevance.

## 4 Materials and methods

### 4.1 Plasmid and strain construction

Strains and plasmids used in this paper are summarized in Supplementary 1. DNA fragments (gBlocks) were synthesized by Integrated DNA Technologies and are listed in Supplementary 2. Oligonucleotide primers were synthesized by Integrated DNA technologies or Sigma Aldrich and are listed in Supplementary 3. Polymerase chain reactions (PCR) utilized Phusion polymerase [New England Biolabs (NEB)] or OneTaq polymerase (NEB) as per the manufacturer’s recommendations.

Restriction enzyme reactions utilized enzymes from NEB following the manufacturer’s recommendations. Golden Gate Assembly mixture was made by reacting equimolar fragments and the desired backbone using T4 ligase (0.5 µL; NEB), buffer (2 μL; NEB), and appropriate type IIS restriction enzyme (1.5 µL; NEB). Gibson Assembly of fragments with >10 bp overlapping arms was conducted as described by Gibson et. al. (2009). DNA was purified using the QIAprep Spin Miniprep kit (QIAGEN), QIAquick Gel Extraction kit (QIAGEN), or QIAquick PCR Purification kit (QIAGEN) as required. Assembled constructs were verified by PCR and/or Sanger sequencing (AGRF) where appropriate.

The transformation of *E. coli* was achieved through the heat shock method using chemically competent cells prepared as previously described (Green and Rogers, 2013) or commercially obtained from NEB (*E. coli* DH5α for routine cloning and plasmid maintenance).

Proteins encoding npAA synthesis, MppJ, MppQ, MppR, MppS, and MppO, were codon optimized for expression in *E. coli*. Each protein was placed in an operon based on its size; RBS were designed (Salis et al., 2009) for maximum expression of their respective proteins and verified using the *De Novo* operon calculator. Homologous regions to the *E. coli* K207-3 (Murli et al., 2003) chromosome were included at the ends of the plasmid construct for *E. coli* genomic integration. Parts to assemble operons were ordered as gene blocks (part 1–7) and amplified individually to include the BsaI type IIS restriction sites for the Golden Gate Assembly. The backbone was amplified from pTargetF (Jiang et al., 2015) with primers HE1 + HE2, where part 1 was amplified with primers HE2 + HE4, part 2 with primers HE5 + HE6, part 3 with primers HE7 + HE8, part 4 with primers HE9 + HE10, part 5 with primers HE11 + HE12, part 6 with primers HE13 + HE14, and part 7 with primers HE15 + HE69. Golden Gate Assembly mix (1 µL) was used to transform *E. coli* DH5α (NEB) under the standard protocol and selected on plates containing spectinomycin (100 µg/mL). The successful assembly of pTOP was verified using primers HE37–HE40 and HE57–HE64. To insert a gRNA region and a terminal homologous region, part 7 was amplified using primers HE75 + HE76, which contain restriction sites for SacI. This fragment was inserted into the purified and digested SacI site of the pTOP plasmid and checked for correct orientation with HE71 + HE72.

pTOP and pCas9 (Jiang et al., 2015) were co-transformed into chemically competent *E. coli* K207 (Murli et al., 2003) and selected using spectinomycin (100 µg/mL) and chloramphenicol (30 µg/mL) and maintained at 30°C. Colony PCR confirmed full genomic integration with primers HE51 + HE52 and HE73 + HE74 by colony PCR. Colonies containing genomic integration were passages to 10 mL of LB media, and 10 µL of 100 mM of IPTG was added to initiate the transcription of a gRNA targeting the origin of replication of pTOP. Cultures were passaged to a fresh 10 mL of LB culture with 10 µL of 100 mM of IPTG and cultured at 37°C to remove the temperature-sensitive pCas9 plasmid creating strain *E. coli* KOP. Cells were passaged once more, and glycerol was stocked.

### 4.2 Growth and culture conditions

*E. coli* K207 and KOP were inoculated from glycerol stocks in triplicate to 10 mL of LB and grown overnight at 37°C with 200 rpm shaking. Overnight cultures were used to inoculate 25 mL o LB test cultures in a 100-mL unbaffled flask to a starting OD_600_ of 0.02 and cultured at 30°C to accommodate possible folding of *Streptomyces* proteins or 37°C for optimal *E. coli* growth at 200 rpm. Samples were taken approximately every 2 hours for OD_600_ growth measurements. Samples were taken in parallel for proteomics, and 1 mL of *E. coli* samples was pelleted, washed once with PBS, and frozen at −20°C for analysis. At the end of the fermentation, 10 ODs of culture were harvested and centrifuged at 5,000 g for 5 min at 4°C. Pellets were washed once with equal volume of ice-cold PBS and centrifuged as described previously. The supernatant was decanted, and 1 mL of methanol was added to the pellets. Samples were frozen on dry ice and stored at −80°C for metabolite analysis.

To increase the flux through the engineered pathways, *E. coli* KOP was cultured with the amino acids, directly contributing to the BmePhe or BhEnd pathways. An overnight culture of *E. coli* KOP was inoculated from glycerol stocks, as mentioned above. Overnight cultures were used to inoculate 25 mL of LB test cultures in 100 mL unbaffled flasks to a starting OD_600_ of 0.01 and cultured at 37°C at 200 rpm. To stimulate the BmePhe pathway, 2 mM of each phenylalanine (as supplement to replace phenylpyruvate) and glutamic acid were added to flasks and cultured in triplicate. For the BhEnd pathway, 20 mM each of arginine and alanine were added to culture triplicates. For the global stimulation of *E. coli* metabolism, 40 g/L of glucose was added to triplicate cultures. At the end of the fermentation, 2 mL (approximately 5 ODs) of cultures were pelleted as above, frozen, and stored at −80°C for metabolite analysis.

As a growth burden was seen for feed cultures with alanine and arginine, *E. coli* K207 and KOP were grown in triplicate in 1 mL of LB in a 24-well plate using 20 mM of alanine, arginine, alanine and arginine, or no feed. The 24-well plate was cultured in a FLUOstar microplate reader at 30°C at 200 rpm, and OD_600_ measurements were taken every 10 min (Supplementary 4).

### 4.3 Proteomics analysis of *Escherichia coli* strains

Proteomics samples were taken in biological triplicates from shake flasks, pelleted, and frozen for analysis. *E. coli* pellets were thawed to room temperature and resuspended in water for a final concentration of 5 µg/µL (250 µg of protein). Cells were heated at 60°C with shaking for 1 h to induce cell lysis before 50 µL of sample (approximately 300 µg protein) was mixed with 50 µL of SDS-lysis buffer solution [10% of SDS, 0.1 M of Tris, 20 mM of dithiothreitol (DTT)]. Samples were heated for 1 h at 70°C and then cooled to room temperature. Cysteines were alkylated by adding 40 mM of iodoacetamide (IAA) to each sample and reacted at room temperature in the dark for 30 min. Samples were sonicated for 10 min to sheer residual DNA before 5 µL of 12% phosphoric acid was added to each sample. Furthermore, 350 µL of S-Trap binding buffer (90% methanol, 0.1 M of Tris) and samples were clarified by centrifugation at 13,000 g for 8 min. Clarified samples (250 µL) were loaded onto S-Trap™ Micro Spin 96-well plates and centrifuged at 3,000 g for 2 min, and the flow-through was discarded. This was repeated with the remaining clarified sample until all of the solution had passed through the column. Columns with samples were washed three times with 300 µL of S-Trap binding buffer, the flow-through was discarded, and the S-Trap plate was moved to a new collection plate. For proteomic digestion, 2 µg of trypsin (2 µL of 1 µg/µL) was added to the top of the column followed by 50 µL of 50 mM ammonium bicarbonate (ABC) at pH 8.0. The plate was spun at 3,000 g for 3 min, and the flow-through was added back to the tops of the column. The plate was incubated at 37°C for 16 h in a damp environment. Peptides were eluted sequentially using 80 µL of 5% ACN, 0.1% of formic acid; 80 µL of 50% ACN, 0.1% of formic acid; and then 80 µL of 75% ACN and 0.1% of formic acid. All eluents were collected in the same collection tube, dried, and resuspended in 20 µL of 5% ACN and 0.1% of formic acid before sample injection.

Digested peptides were analyzed by liquid chromatography–mass spectrometry (LCMS). Initially, 2 µL of sample was loaded onto the Thermo Fisher Acclaim PepMap C18 trap reversed-phase column (300 μm × 5 mm nano viper, 5 µm particle size, 100 Å pore diameter) at a rate of 15 µL/min. Peptides were separated with mobile phase solutions of H_2_O (solvent A) and 80% of ACN (solvent B), both with 0.1% formic acid onto a Waters nanoEaseTM M/Z CSH C18 resolving column (130 Å, 1.7 µm, 300 μm × 100 mm) at 40°C using a 60 min gradient of 8 to 95% acetonitrile in 0.1% of formic acid at 3 µL/min flow rate. The eluted peptides were run on an Orbitrap Exploris Mass Spectrometer (Thermo Fisher, United States). The MS parameters were for full MS at a resolution of 120,000, with a scan range of 340–1,110 m/z.

Data analysis was performed with Spectronaut (Bruderer et al., 2015) version 15 using direct DIA and default settings. The *E. coli* BL21 genome (accession number: NZ_CP053601; closest annotated genome) with the manual addition of protein sequences from MppR, MppO, MppJ, MppQ, and MppP, was used as the reference library. A 1% false discovery rate was applied for cutoffs at the peptide spectral match, peptide, and protein group levels. Quantitation was performed at the MS2 level with Q-value filtering <0.05 and *p*-value filtering <0.05. The mass spectrometry proteomics data have been deposited to the ProteomeXchange Consortium via the PRIDE (Perez-Riverol et al., 2022) partner repository with the dataset identifier PXD056235.

### 4.4 Metabolite analysis

#### 4.4.1 Amino acid extraction

Frozen cell pellets were thawed on ice and transferred to 5 mL of screw cap tubes, and approximately 200 µL of 0.5 µm glass beads (11079101; BioSpec Products) were added. The bead beater Precellys 24 (Bertin Technologies) was pre-chilled to −1°C using ethanol and dry ice. Samples were pulsed at a speed of 4.00 m/s, with 5 × 45 s pulses, 30 s interval, and max tube speed of 8. To purify the metabolites, 1 mL of phenol:chloroform:isoamyl (25:24:1) was added to samples and vortexed to mix. To separate the organic layers, samples were centrifuged at 16,000 RCF for 5 min at 4°C. The aqueous layer was transferred to a new 2-mL Eppendorf tube, being careful not to disturb the interface layer containing proteins. Samples were evaporated in a rotary evaporator at 30°C until the sample volume was approximately 400 µL to remove most of the methanol. To further dilute any residual methanol, 1 mL of water was added to extractions. Samples were freeze-dried overnight and then reconstituted in 100 µL of 2% of ACN.

#### 4.4.2 Generation of non-proteinogenic amino acid standards

To generate a standard for npAAs β-methylphenylalanine and β-hydroxyenduracididine, a purified mannopeptimycin-α standard was acid-hydrolyzed. Purified mannopeptimycin-α was provided by Zoetis. A sample containing 1 µg was reacted with 200 µL of 6 N HCl at 110°C for 24 h. This reaction was dried under N_2_ before 20 µL of NaHCO_3_ was added to neutralize the reaction. The sample was diluted to 100 µL with 2% of ACN before injection into the LC–MS.

#### 4.4.3 LC–MS/MS of intracellular metabolomics

For quantification, samples were diluted 1/100 with 2% of ACN, and 10 µL of each sample was injected for analysis on a Shimadzu Nexera HPLC system coupled to an 8060 MS/MS and separated on a Shim-pack Scepter PFFP-120 (3 μm, 2.1 × 150 mm) with a flow rate of 0.3 mL/min. The mobile phases used were 0.1% of HFBA, 0.05% of FA in water (A), and 0.1% of FA in acetonitrile (B). Gradient conditions are as follows: 100% A for 3.5 min, increased to 98% B from 3.5 to 9 min, isocratic for 3 min, returning to 100% A within 0.1 min, and holding at 100% A until 15 min. Single ion monitoring (SIM) was conducted at a micro scan width of ± 0.3 amu on the positive ionization mode. Initial spectral scans were run within tightly defined mass windows to capture the molecular weights of desired products: BmePhe, m/z = 179.82–180.18; enduracididine, m/z = 172.83–173.17; BhEnd, m/z = 188.81–189.19. MRM methods were developed for BmePhe with a precursor mass (Q1) of 180.10 m/z and MRM tracking of fragments 134.10, 117.20, and 163.35 m/z on the Q3. For BhEnd, a Q1 mass was set at 189.10 m/z with MRM fragments of 84.15, 114.15, and 68.10 m/z on Q3. These fragment ions were verified against the CFM-ID 4.0 online tool (Wang F. et al., 2022). Enduracididine was in too low of a concentration in the standard to create an MRM method; however, fragment fingerprint patterns were used to confirm the presence of the compound in strains (Supplementary 5). MRM transitions for proteinogenic amino acids were optimized using authentic reference standards purchased through Sigma (LAA21).

## Data Availability

The mass spectrometry proteomics data have been deposited to the ProteomeXchange Consortium via the PRIDE partner repository, accession number, PXD056235. Available at: https://proteomecentral.proteomexchange.org/cgi/GetDataset?ID=PXD056235
