# Supplementary material for: Biosynthesis of novel non-proteinogenic amino acids β-hydroxyenduracididine and β-methylphenylalanine in Escherichia coli
Source: Front Bioeng Biotechnol. 2024 Oct 9;12:1468974. doi: 10.3389/fbioe.2024.1468974 (PMC11496134; doi:10.3389/fbioe.2024.1468974)
Supplement: Supplementary file 1 [file DataSheet1.docx]

Supplementary:

Supplementary 1: Strains and plasmids used in this study.

| E. coli strain | Description | Source/reference |
| --- | --- | --- |
| DH5α | *lacZΔM15*, *recA1* | NEB |
| K207-3 | F*‐ompT hsdS_B_(r^‐^m^‐^), gal dcm* (DE3), *panD::panDS25A,*Δ*prpRBCD::*T7prom*‐sfp,* T7prom*‐prpE, ygfG::*T7prom*‐accA1‐*T7prom‐*pccB* | Murli *et al*. (2003) |
| KOP | K207-3 *mppP mppQ mppR mppO mppJ sm^R^* | This work |
|  |  |  |
| Plasmids | Description | Source/reference |
| pCas9 | *repA101*(Ts) *kan P_cas_-cas9 P_araB_-Red lacI*^q^ *P_trc_*-sgRNA-*pMB1* | Jiang *et. al*. (2015) |
| pTargetF | *pMB1 aadA* sgRNA | Jiang *et. al*. (2015) |
| pTOP^a^ | pTargetF sgRNA- DS*nhaR trunc.nhaR (*292 bp*) P_J23100_ mppP mppQ P_J23100_ mppR mppO mppJ aad trunc. SR36 (*300 bp) | This work |

^a^ truncated nhaR and SR36 were used as homologous arms for genome integration after Cas9 DSB.

Supplementary 2: Gene blocks used in the study. Green indicated gRNA sequence.

| Part 1-  ggctacggtctcacaaattgcttaactggtttaactcccagggattaaacgtagaaatcctcggcgagtttgatgatgccgctttgatgaaagcttttggtgcgatgcacaatgcaatcttcgttgccccaacgctttatgcatatgacttttatgccgataaaactgtcgtagaaattggtcgcgtcgagaatgtgatggaagagtaccatgctatttttgctgagcggatgattcagcacccggcggtacagcgaatctgcaatacggattattctgcgctttttagtccagcggtgcgttaaaccgatacaattaaaggctccttttggagcctttttttttggagattttttgacggctagctcagtcctaggtacagtgctagcggactcattattacaaaggggtatcatgtcggggacacagcaggtaaaggcagctttaggagattccgaaggagataccggcaatcttacacagttagagttcttggcgcttaatagtgagtttaatattgcggatggacatgcgcgtcaggccttaaccccagggcaatccaagattgttgatgatttaccgctgttgtttgctgagggggagaagcgcccagtagaggagcttgaacgtgaggctcaccatgccttctttaccgcacttggccagcattcgtacccttcagcgcctgggcgtgtcttatcttgttattccagtagtgtggcgatggagattttgagccgctccttgagcgaaacaattgagtctgtcgctctggtacaccctacgttcgataatattgcagacttgctgcgcggaaatggattgaaattggtgccattggccgaggatcctttacacggtgacgatctggacgtaagtctgttgaagagtgtgggctgtgtcttcctgactacccccaacaacccgactggcaaggtcgtttcgcgcgaacgccttagagaccgtagcc |
| --- |
| Part 2-  ggctacggtctctccttacgcgtcttgccgagcagtgcgctgaacatggggtcattctggctctggatacctcttttcgcggcttcgacacgcgtgcgcattatgaccactacgaagttttgaacgcaagtggagttcgttgggtagttattgaagacacgggaaaactttggcctaccttggatttgaaagtaggaatgctggtccacagtgaaaacttagcattgcctgtagagaagatttattcagacattctgctgggagttagcccgcttatcctggcaatggttcgccgtttctccgaggatgcggcagcaggcggtcttgaggaccttcatcgtttcattgcggcgaatcgtgccatggtccgagctgagttggcgggattacctggcgttactgtccccgatccggactcacgcgctagtgttgaacgcgtcgcaatcgacgatttaacgggcactcaggtatgggctaaattacgtgagcacaatgtctacgccttaccatgccgcccttttcattgggcgaacccgtcggaaggggatcatacgcttcgtcttgcgttagctcgctccactgatccattagcgcaatctgtccgcgcactgcgtcacgttctgaaacagcgttgatgatttcaggcgaaataaggattaaggtatgactcctgttgcggagggtggtttgcctcatggttcggtcccctctcttagtcacacgcgacagtggaggccaggggtagtgcaagaagtagctccggctggggtccttgaccttggtcctggttacatcgagcccgctttgcttcctgttcgtttattgcgtggcgcctatgaacaagctctggcggaatacggtgccgccgcattagggtatggacacgacccgggtgctcaacctctgcgggaccggttggctgctcgtgctgcggcggcggatgggttaccttgcgaccccgatcaggtcctgttgagaccgtagcc |
| Part 3-  ggctacggtctcactgttgacgagcgggacttcccaggctttgtatcttctggcaacctctttggccgctccgggcgatacagtactgactgaagaactttgctacgatcttggacagcgtatcttccgcgactgttcccttcgtttgcgccaagtcgcaatggacggatcagggatgctgccagacgctttggatcgcgccttaactgagggcgcgcgtgcgggcgctaaaacagcatttgtatatctgacgccgactcaccacaatccgaccggccatacaatgccactggctcgacgtcgtttactgcttgaagtagctgcacgccatgacgtgcttatcgttgaagatgacgcctacacggagttgtcattgattccagaccgcactccgccgccaagcttggcggcattagctggctaccgccgcgttgtccgtctgtgtagtttcagcaaaacgttgggtccaggattgcgcctgggttggttgttggccgatcgcgaactggcgggacgtcttgcgacacatggtctgttcgtatccggcggtagcttgaatcacacgacatctctggccgtttccaccctgttggcaagcggggcctatgatcgccacctggatgccttccgggcccaattgcgggctaggagagatgcattggttggtgcgctgagagcgatgttggatgacggtgtcgagttgcgcacaccagagggaggtttttttttatggctcagagcaggcgacggagcagatgagcgtgagttactggatggtgcggctcgcgcaggcgttagaattgcggcgggctcgcgtttcggcacgacccagggcgcgggactgcgcctggcattttcgtttaacccgccggcattgttagagcaagctgcgaagcgccttacaactgcttggtctggttcaacccctgatttggagatcggagttcgcagctgaataatgagaccgtagcc |
| Part 4-  ggctacggtctcaataaaaaacgcccggcggcaaccgagcgttctgaattgacggctagctcagtcctaggtacagtgctagctcgtagatatagaggaggattctgaatgaccactagcacgggtacgaacgggcgtcacacagttgctggcccgggcagcgcagggcctgtaggttattcattaccactgtcaccgacaggcgagtcggccatgctgactccaccaccctggcacttctccggagaagtagtaatggtagattatcgcgtagatcccgatgcagctcgtcgctttcttcccccggggttggaaccgggtgcggatcctggtgcggcagctgcggtatttgcaacctggcagtggtgtagtcaggatggagctgaattgacagatcccggccgttgccaattcggagaattcttgatcttactgtcgtgcgaattcgagggtcgccctatggcacgctgcccttacgcctgggtcgatcaagcggtcccaatgatgcgcggctgggtgcaagggatgcccaaacagtttggagttattcatcagtcccgccccgtcactgtaggtaaagcaggttcccgtcttgcaccgggtggacgctttgacggcgctttgagtgtgcatggacgtcgcgtcgttgaggctagtgttacggtcgatcgctcaacagatcaaccgcctgcgcttcatgacgtcccgctggcacacacccttgtctttccagaatgggttccgtctggtgggggtcctcgcccgagattagttgcttccgaggtttcagatgtagagtttagccccatttggacaggttcgggtgacttaactttctttgatggtcttggcgatgacttcggtgcattggctccattggaggttggaagcggccacgttttctcgtacggtgagacgctgcatggtggtcgcttgctgtctgattattcagttagcaacgatatctcacatgagaccgtagcc |
| Part 5-  ggctacggtctcacacatgaccacggtggacaatctgcagaagatccattacattcctgatgactttacacatatataagggggatttatgctgacccttcacttacaagatgacgatgtcgcggcaattgacgcggtagccgacgaactgagtcgccgttacgactccgtggaaagcactgaatttcaagcggagagtcgcctttacgccgacgaattaccgcgccgagttcggcgagcgctgcacgagtatcgctctacggagaaatcagggatcttggtcgtgacagggcttccggttgacgattctgcattaggggcgactcctgcggatcgtcgtcacaaaccagtaccaagcacatcgctgcgtcaggatattgcgttctatttgatcgcaaaccttctgggagatccaatcggctgggcaactcaacaggatgggttcatcatgcacgatgtatatcctgtccaaggatttgaacacgaacaaatcggttggggttccgaggaaaccttaacttggcatacggaggacgcgtttcatccgttgcgtacagattaccttggattgatgtgcttacgcaacccggatggggtggaaacaaccgcgtgtgatatcgcagatgtggagattgacgatgaaacccgtgagactctttcccaggagcgctttcgcatccttcctgacgacgcccaccgcattcatgggaaagctccaggtgacgaatctgctcgtgaatccgccctgcgtgaacgcagccgtcaacgcgtagctagtgccttggaaagtcctgatcctgtcgccgtcttatttggggatcgcgatgacccctatttgcgtatcgacccgcactatatgcagggagtgcagggcgaaacggaacaacgcgctttagaaaccatcggagcagctattgacgatgcaatgagcggtgttgttcttagtccgggtgacatcgtcttcattgataactaggagaccgtagcc |
| Part 6-  ggctacggtctccactatcgcgtcgtacacggccgcaaaccgtttcgcgcgcgttttgacggcacggatcgctggcttcgtcgcttgaatatcgctagagacttgcgcaaatcacgcgaagcgcggttggcggcgacaacccgcgtaatttattgatgaccaggcccagcagggaggtcattccatgtccacagaagtctcagaagcccaggcgcgtcgtgcagtcgctgatatctttaattccaccttggcatcttccgctattggcgcggcatgggagttaggggccttagacgagttacgcgaaaacgggaagttagatgtgtccgatttcgccgtccgccatgatcttcatgaaccggctgtagtgggtatgtttacggcactggcttcggttggtattgttcgccgcgagggggcaacagtggtcgtaggtccttacttcgatgaagccaatcatcaccgtagtctgttccattggcttaaccaaggctctggagagctgttccgccgcatgcctcaagttctgccgaacgaaaaccgcactggtaagttttaccaacgtgacgctggcgcaatttcctatgcctgtcgtgaaatctcagagcgctattttgatccagccttctgggccgctgttgacgggttaggctatactccgactacggtggctgatcttggctcaggatccggtgagcgcttaatccagattgcccgtcgctttccaggcgttcgcggtctgggtgtggatattgccgacggcgcgattgcaatggctgaaaaagaagtagcagcgaagggatttggggaccaaatcagtttcgtgcgaggagacgcgcgtactatcgatcaagtctccgcgcgcggtgagttcgcagaagttgatctgcttacttgcttcatgatgggtcacgatttttggccgcgtgaaaactgtgtacagactttgcgtaaactggagaccgtagcc |
| Part 7-  ggctacggtctccaacttagggctgcattcccgaatgtccgccgcttcctgctgggagatgctacgcgtacggtcggtattccggacagagagctgccagtttttacattaggcttcgagtttgggcatgatatgatgggagtgtacttgcccaccctggatgagtgggacggcgttttcgaggaaggcggatggcgttgcgtgaagaaacatgctattgatagcctttcggtcagcgtagtctttgaacttgaataaagagaatataaaaagccagattattaatccggcttttttattatttttattgatagtgttttatgttcagataatgcccgatgactttgtcatgcagctccaccgattttgagaacgacagcgacttccgtcccagccgtgccaggtgctgcctcagattcaggttatgccgctcaattcgctgcgtatatcgcttgctgattacgtgcagctttcccttcaggcgggattcatacagcggccagccatccgtcatccatatcaccacgtcaaagggtgacagcaggctcataagacgccccagcgtcgccatagtgcgttcaccgaatacgtgcgcaacaaccgtttgacagctagctcagtcctaggtataatactagttatggaaacaatgtctcaaggttttagagctagaaatagcaagttaaaataaggctagtccgttatcaacttgaaaaagtggcaccgagtcggtgcctgtaacagagcattagcgcaaggtgattttgtcttcttgcgctaattttttttgaggagaccgtagcc |

Supplementary 3: Primers used in the study.

| HE1 | ggctacggtctcagttcatgtgcagctccataag |
| --- | --- |
| HE2 | ggctacggtctcatttgcgccctaagggataacgcaggaaagaac |
| HE3 | ggctacggtctcacaaattgcttaactggtttaactcccaggg |
| HE4 | ggctacggtctctaaggcgttcgcgcgaaacgac |
| HE5 | ggctacggtctctccttacgcgtcttgccgagc |
| HE6 | ggctacggtctcaacaggacctgatcggggtcgcaaggtaac |
| HE7 | ggctacggtctcactgttgacgagcgggacttc |
| HE8 | ggctacggtctcattattcagctgcgaactccg |
| HE9 | ggctacggtctcaataaaaaacgcccggcggcaac |
| HE10 | ggctacggtctcatgtgagatatcgttgctaactgaataatcagacagcaagc |
| HE11 | ggctacggtctcacacatgaccacggtggac |
| HE12 | ggctacggtctcctagttatcaatgaagacgatgtcacccggac |
| HE13 | ggctacggtctccactatcgcgtcgtacacg |
| HE14 | ggctacggtctccagtttacgcaaagtctgtacacag |
| HE15 | ggctacggtctccaacttagggctgcattcccg |
| HE69 | ggctacggtctcagaacctctttattcaagttcaaagactacg |
| HE37 | tgagcgtcgatttttgtgatgct |
| HE38 | gcgatcaccgcttccctc |
| HE39 | ttctttgatggtcttggcgatgac |
| HE40 | gtcatcgccaagaccatcaaagaa |
| HE57 | agaaggcatggtgagcctc |
| HE58 | cctgactacccccaacaacc |
| HE59 | caaagcctgggaagtcccg |
| HE60 | gccggcattgttagagcaag |
| HE61 | cagcgctcgccgaactc |
| HE62 | aggtgacgaatctgctcgtga |
| HE63 | cggcccagaaggctgga |
| HE64 | tttggccgcgtgaaaactg |
| HE75 | atcgtgcgagctcagagaatataa |
| HE76 | ggttttttggagctcagtcgtc |
| HE71 | tgggttcaaaaaccatgattacgc |
| HE72 | Ctaactacggctacactagaaggac |


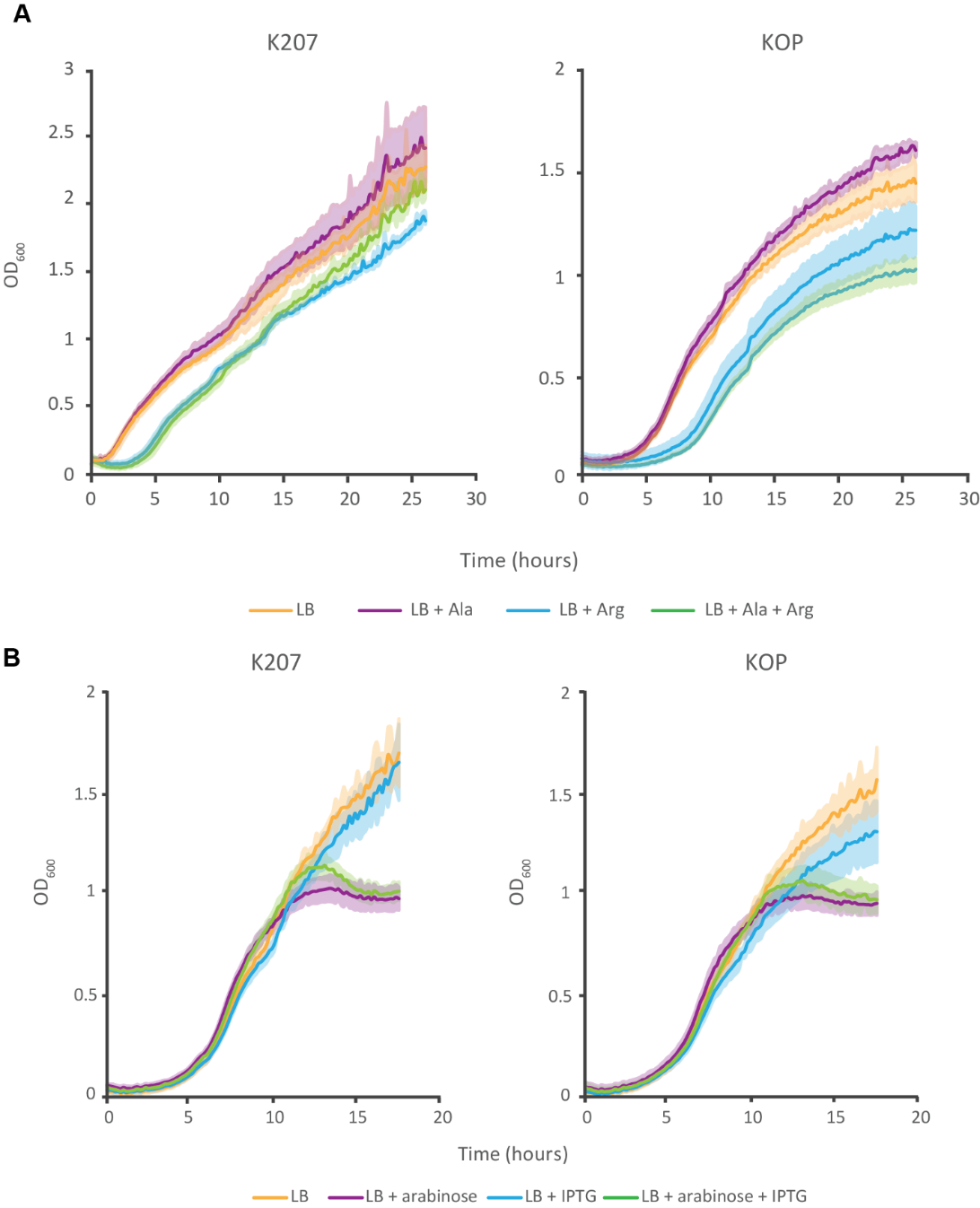

*Supplementary 4: Growth curves of* E. coli *K207 and* E. coli *KOP in the presence of arginine and alanine or inducible molecules. Comparative OD measurements with 20 mM each alanine, arginine or both. Samples were taken in triplicate and each point represents the mean +/- the standard deviation.*


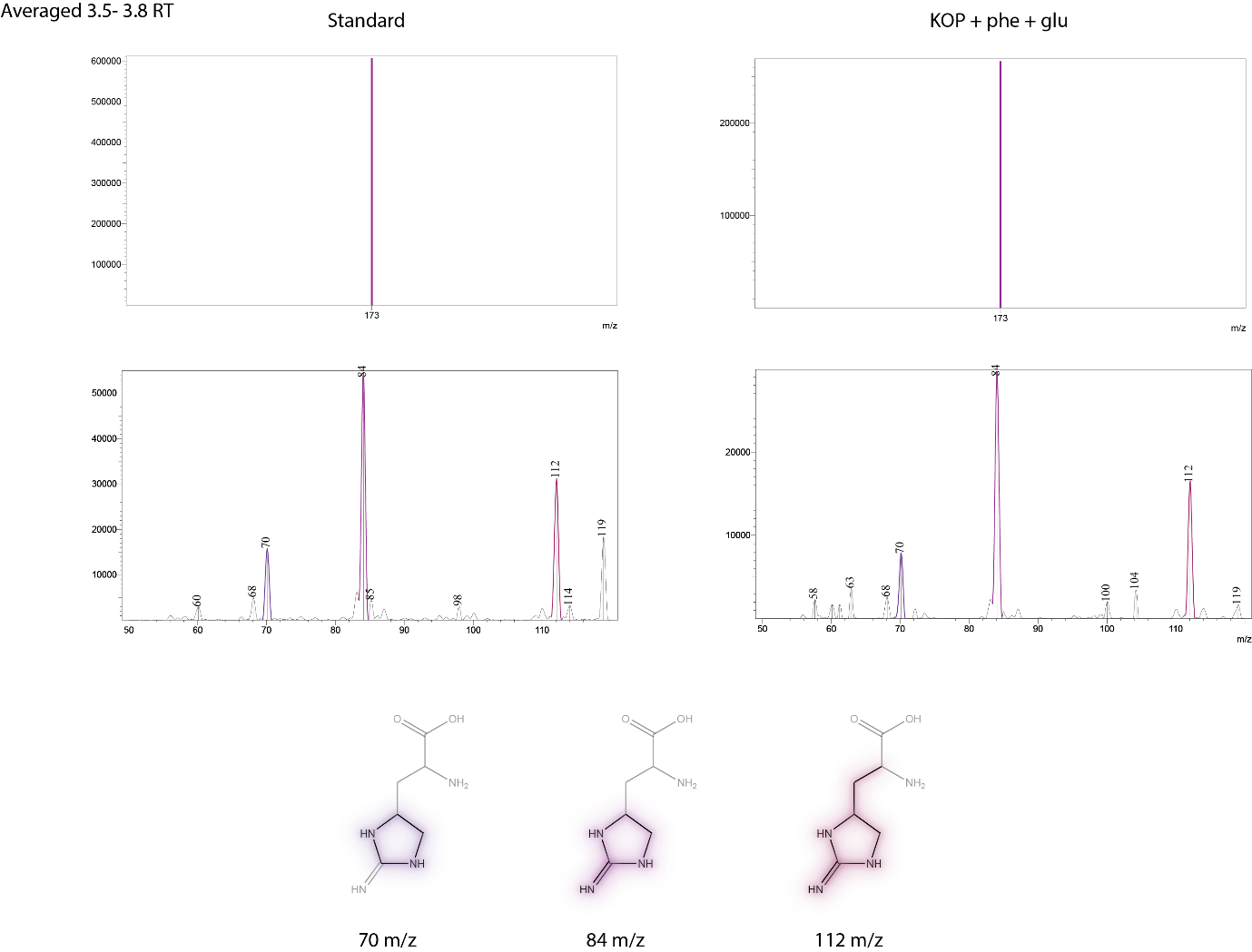


*Supplementary 5: Daughter fragment patterns for enduracididine in cultures against the standard with corresponding structure predictions.* E. coli *KOP cultured with 2mM phenylalanine & 2 mM glutamic acid was taken as a representative fragment pattern.*
